# On the Medicinal Efficacy of Sulphuric Acid

**Published:** 1800-12

**Authors:** Robert Kinglake

**Affiliations:** Chilton super Polden


### *On the medicinal Efficacy of Sulphuric Acid.*

To the EDITORS of the MEDICAL and PHYSICAL JOURNAL.

Gentlemen,

CHRONIC eruptions on the cuticular furface are well known to form a frequent, and occafionally a very diftreffing malady; they alfo affume various appearances, prefent different degrees of inveteracy, and often prove irremediable under the moft approved mode of treatment. This practical difficulty has rendered me invariably pufillanimous in my opinion of the iffue of thofe complaints; and experience of the infufficiency of the ufual practice, has obliged me fucceffively to combat the diforder with different agents, before even an apparent cure could be effected, which, in the majority of inftances, has been ultimately evinced to have been a temporary fufpenfion only, and not a real extinction of the difeafe. The warm bath, the internal and external ufe of a folution of the corrofive muriat of mercury, antimonials, guaiacum, &c. have, feparately and conjointly, failed in producing the defired effect. Nor has the nitrous acid, under my direction, juftified the reputation given to it, or in any inftance proved permanently falutary. The chemical explanation which has been recently given to the medicinal efficacy of various oxydated and oxygenated preparations, particularly the oxides of quickfilver, by afcribing the agency exerted to the oxygene in combination; and alfo the popular efteem in which fulphur is held againft eruptive diforders, induced me to give an extenfive trial to Sulphuric Acid, as probably poffeffing, in concentrated combination, the feparate powers of its conftituent principles. It is with heart-felt fatisfaction that I have now to detail how far my expectations have been realifed.

1. A child, about three years of age, had been upwards of twelve months affected with eruptions, which originated on the face, and were foon fpread over the cuticular furface, rather in partial clufters than in equal diftribution. This eruption had its ftages of rife, progrefs, and declenfion: It began with an inflammatory afpect, accompanied with preternatural heat and itching, and terminated in a furfuraceous difquamation. This courfe was ufually completed in about a fortnight, and refumed at fhort but irregular intervals. Various medicines had been exhibited, but what they were could not be diftinctly learned; no lafting relief, however, had refulted from them.

them. This patient was directed to take ten drops of the ſulphuric acid in a tea-cup full of pure water three times a day, and to have the eruptions waſhed as often with a ſolution of the ſame acid in water, in the proportion of one drachm of the former to one pint of the latter. This was performed by dipping a ſmall piece of linen in the liquid, and moiſtening with it the parts affected. The external application was at firſt productive of ſevere pain, but this inconvenience daily diminiſhed. The drops reſted well on the ſtomach; in the courſe of a week much amendment was apparent, the eruptions were much ſubſided, and felt and appeared hard and ſcurfy, but the intermediate ſound ſkin continued highly reddened. The curative progreſs advanced uninterruptedly, and in three weeks from the commencement of the treatment, the little patient was comparatively well; the blotched relicks alſo ſoon became conſiderably effaced; and at this time, now upwards of four months, the child remains perfectly free from complaint.

No other medicine was adminiſtered during the employ of this remedy; indeed, the general health was ſpeedily raiſed from a very languid and irritable ſtate to a more robuſt and tranquil condition. The appetite became craving; the ſeveral evacuations were regular; and that by cuticular perſpiration was ſenſibly increaſed. To this latter circumſtance may be attributed the little inconvenience induced by the inflammatory redneſs excited on the ſkin by the lotion; the increaſed evaporation of perſpirable matter transferred redundant heat, and ſalutarily refrigerated the preternatural temperature.

2. A man of a robuſt temperament, aged about 30 years, by trade a ſhoe-maker, had been for ſix months grievouſly afflicted with an excoriation of almoſt the entire cuticle of both legs. In ſome parts, ſmall, deſtructive ulcerations had reached the muſcular ſtructure, and a morbidly decompoſing proceſs appeared to be ſpreading in every direction over the affected limbs, yielding a vaſt diſcharge of thin fœtid fluid. This complaint was formally limited to the lower extremities, but a ſympathetic itching was propagated over the whole cuticular ſurface. The general health had not been ſenſibly affected. Thirty drops of ſulphuric acid were directed for this patient, to be taken in half a pint of cold water, gradually extending the doſe to one tea-ſpoonful, if not contra-indicated by any forbidding occurrence, ſuch as nauſea or purgation. A ſulphuric acid lotion, of the ſtrength before-mentioned, was alſo ordered to be applied by means of a bandage, wetted in the liquid, and rolled tightly, by equal and progreſſive rounds, from the feet to the knees. The drops produced no inconvenience; the lotion induced exquiſite pain; but this appeared to be the effect of of an impreſſion that had promptly deſtroyed the diſeaſed action; a high degree of inflammatory redneſs and tenſion enſued, but which was readily controlable by the application of cold water. A purely aqueous fluid, (coloured for the ſake of diſguiſe) was now ſubſtituted, in which the bandages formerly wetted in the ſulphuric acid lotion, were ordered to be moiſtened, and applied in a ſimilar manner, renewing them as ſoon as either dry, or a ſenſe of diſeaſed heat recurred.

This procedure diſſipated the tenſion, pain, and redneſs within two days, and its continued uſe, conjoined with the gradually augmented doſes of the acid, during about ten days, nearly effected a cure. A dry ſcurf on the legs exhibited the only remaining veſtige of this inveterate diſeaſe.

The ſmall ulcerations, as before remarked, on different parts of the legs ſpeedily changed their ill conditioned aſpect, and healed with rapidity.

The ſulphuric acid was in this caſe taken to the extent of two tea-ſpoons full three times a day, conjoined with a ſmall proportion of opium, to obviate a purgative effect which it had previouſly produced. It is worthy of notice, that at one period this patient was highly ſalivated by the acid, the pulſe alſo was quickened ſimilarly to what uſually occurs under the higheſt ſyſtematic operation of mercury. This effect, however, ſubſided much more ſpeedily on ſuſpending the acid, than ordinarily happens when reſulting from mercury. Three days diſcontinuance of its uſe was followed by an entire ceſſation of the ptyaliſm. The relief obtained in this caſe was a complete removal of morbid action, that had advanced to an ulcerative proceſs, and which appeared to be moſt virulently circumſtanced. It had reſiſted the ordinary attempts of regular, and the extraordinary endeavour of irregular practice.

Two months are now elapſed ſince the commencement of this treatment; the affected parts continue perfectly well, with the exception of occaſional itching and a ſenſe of morbid heat, which have at length been nearly ſubdued by plunging the legs, every morning and evening, in cold water as high as the knees, keeping them in that ſituation about one minute, and then rolling them as before-mentioned with a bandage ſqueezed from cold water.

This ſudden and conſtant mode of reducing the diſeaſed heat, promiſes ultimately to correct the vitiated proceſſes on which its generation depends, and to re-eſtabliſh the ſalutary temperature.

3. A ſtout yeoman, aged about 25, requeſted my advice for an obſtinate eruption on his right arm, which had exiſted about two months. Its origin was confined to a few confluent puſtules, tules, which fpeedily fpread, by contiguous extenfion, over the whole upper furface of the arm. At the time of his firft application, this puftular affection had a very loathfome and inveterate afpect; its general fuperficies was much above the level of the cuticular furface, and its centre was elevated into a fuppurative protuberance, from whence oozed, as from other parts of it, a thin fanguineous fanies, not ftrikingly offenfive.

In this cafe the fulphuric acid was employed both internally and externally, as before fpecified, with the difference only of reducing the quantity of the acid in the lotion to the proportion of half a drachm to one pint of water. This alteration was a meafure of precaution, taught both by the pain induced in the former cafe, and the highly difeafed temperature prevailing on the whole arm. No fort of inconvenience arofe either from the internal or external ufe of the acid thus managed, and within three weeks the patient was difmiffed perfectly cured. Complete difquamation of the unfightly groupe of puftules had occurred, and the fubjacent cuticle was fmooth, foft, and flexile.

4. A young woman, under the preffure of much conftitutional debility and morbidly high temperature, had been nearly eighteen months incommoded by large diftinct eruptions on different parts of the body, appearing and difappearing at irregular intervals; a large puftule on one of the legs at length ulcerated, and fpread by rapid decompofition of the contiguous parts to great extent and confiderable depth; the attendant irritation was extremely acute, and marked by a high degree of furrounding tenfion, inflammation, and even vefication; the fenfe of heat was, to ufe the patient's term, fo intenfely burning, as to leave no doubt of exceffive temperature conftituting the chief fource of the diftrefs. Conformably to this opinion, I directed it to be reduced, by enveloping the limb in a cloth wetted with cold water, and renewed every half hour. The advice was fedulioufly purfued, and the alleviation in the courfe of the fame day was incredibly great and durable; the fenfe of burning was exchanged for grateful warmth. On the fubfequent day, the altered afpect of the limb ftrikingly evinced the falutary reduction of heat which had been effected, but the ulcerated furface continued of a dark mottled hue. The fore was now directed to be dreffed every morning and evening (after wafhing it with cold water) with an ointment compofed of half a drachm of fulphuric acid and one ounce of hog's lard, and the leg to be afterwards bound from the foot to the knee with a fuitable bandage dipped in cold water. The fulphuric acid was alfo taken by progreffive augmentation, as in the former cafes, and to an equal extent, but without any affection of the falivary glands enfuing from it.

The falutary effects of the treatment in the patient under confideration, even exceeded any thing that ever occurred to me to obferve from the moft efficacious remedies. Within three weeks this highly diftempered young woman was unfettered from her noifome malady, walked to my houfe, (a diftance of three miles) without halting, fatigue, or an eruption, and with no other trace of her former corroding ulcer, than a fuperficial cicatrizing fore, of very inconfiderable dimenfion, and which I have fince learned is now perfectly healed.

Among feveral other inftances of fubordinate importance, I may relate two others, which have as unequivocally, in my eftimation, eftablifhed the fuperior efficacy of the fulphuric acid, in the cure of cuticular eruptions, as either of thofe which have been recited; but a more extenfive narrative would occupy more of your time and room than can be reafonably prefumed on. If what has been advanced in favour of the particular curative powers of this remedy fhould be deemed fufficient to entitle it to a full inveftigation of its poffible efficiency, my object in fubmitting to public confideration the fuccefs of my experience will be amply accomplifhed. It may, however, be proper to add, that the falutary influence of the treatment defcribed has been fo uniformly apparent in all the cafes in which it has been employed under my direction, that it remains for me yet to learn an exception to its efficacy. It may be obferved, that in the management of the preceding cafes, the prevailing temperature, as indicated by the *patient's sensations*,* was a leading object of attention. This regard proceeded from an opinion which has been long indulged by me, that all eruptive diforders and ulcerative proceffes, in common with every variety of febrile affection, are effentially connected with motions of the animal principles of life, that generate exceffive heat, which in its turn produces morbid actions and fenfations. Confonant to this doctrine, I have attempted the cure of eruptive complaints by the external application of cold water and the refrigerating influence of increafed perfpiration, and in fome inftances effects highly beneficial have by this mode been produced; but on moft occafions the *habit* of morbid action has not vanifhed with the reduction of the difeafed temperature: hence an agent appeared to me to be wanting, that by affecting the exifting

---

* The feelings of the patient, with refpect to temperature, have ferved me as a much more accurate guide than the indications afforded by thermometrical menfuration. This feeming preference, however, may not be well founded, but be afcribable to my thermometer not poffeffing fufficient mobility to exhibit, with fuitable precifion, the diverfified range of the prevailing heat.

exifting affinities which hold the principles of animal ftructure in vital combination, a falutary change might be induced from thofe morbid conditions of motive power, that at once conftituted and actuated the prevailing difeafe.

The fulphuric acid has hitherto proved, in my experience, fully competent to this effect; but whether its efficacy be due to the fpecific chemical virtue of its oxygenous principle, its conftituent combination, or to its more general ftimulant properties, is exceedingly problematical.

No fufficient documents have yet been furnifhed, either experimentally, inductively, or analogically, on which might be fatisfactorily refted an explanation, either how medicinal impreffions are made, or how the power imparted is incorporated and appropriated. The recondite operation of the animal œconomy, in converting and affimilating matter, and thereby generating fpecific power and efficiency, is yet but dimly perceived by *Chemical Phyſiology*, which, unqueftionably, conftitutes the only legitimate mode of refearch. Extenfion of facts promifes progreffively to illuftrate the view, and ultimately to unveil the latent fimplicity of native truth. I am,

GENTLEMEN,

Yours, very refpectfully,

ROBERT KINGLAKE.

*Chilton super Polden,*
*Nov.* 12, 1800.

P. S. In your laft Number is a Comment on a former Paper of mine, relative to Parturition and the Delivery of the Placenta,* by a Mr. S. THOMAS. I muft be very brief in anfwer to an opponent, who appears to have attacked my opinion without examining or regarding the grounds on which it refts. He has fummarily replied by fufpecting my *practical familiarity* with natural labour, though the doctrine advanced in the paper in queftion, is diftinctly ftated to have been fuggefted by *ample experience.*

It muft, on reflection, occur to your correfpondent, that fuch unqualified diftruft is a breach of decorum, equally incompatible with focial urbanity and philofophical argument. I will only trouble you to make known to Mr. S. Thomas, that my opportunities for practical information have been fufficiently extenfive and diverfified, to warrant me in exercifing an independent judgment on the fubject of parturition; that I might alfo boaft of having duly profited by the tuition of the preceptors whom he has cited; that, were it neceffary, innumerable examples might be adduced of *fpontaneous parturition*, without a fingle

* See Medical and Phyfical Journal, No. XVIII. p. 156.

a fingle inftance of *perineal laceration*; and, finally, that my wifh is not, as Mr. S. Thomas gratuitoufly fuppofes, to abandon the practice of midwifery to uninformed females; but to vindicate obftetric philofophy from the imputation of recommending the *uniform neceffity* of manual affiftance, and to reftrict its fcientific authority for artificial aid to the comparatively rare accidents of unnatural prefentation and other extraordinary occurrences.

